# A discussion on the potential impact of residential radon exposure on the quality of exposure and risk assessment for former uranium miners

**DOI:** 10.1007/s00411-020-00875-6

**Published:** 2020-10-08

**Authors:** Jing Chen

**Affiliations:** Radiation Protection Bureau, Health Canada, 775 Brookfield Road, Ottawa, ON K1A 1C1 Canada

**Keywords:** Radon-222, Risk assessment, Uranium miners, Exposure assessment

## Abstract

Epidemiological evidence of lung cancer risk from radon is based mainly on studies of underground miners where occupational exposures were, historically, relatively high in comparison to residential indoor exposure. However, radiation protection measures have caused radon levels in uranium mines to decrease significantly in more recent periods. Miners’ occupational exposure is limited to their working years while they are exposed to environmental radon at home over their entire lifetime. Even during their limited working years, workers spend much more time at home than in workplaces. The biological effect of radon in mines cannot be distinguished from the biological effect of residential radon. Therefore, for an exposure–risk relationship study of former uranium miners, excess radon-induced lung cancer cases should be related to the combined radon exposure cumulated in workplaces and at homes in excess of the radon exposure of the reference population. This is especially important when residential radon levels differ or vary significantly between miners and the reference population over the course of extended follow-up years. This paper reviews some recent studies on former uranium miners, shares what seems controversial to the author and wonders whether lifetime exposure at home to widely varying radon concentrations can actually impact the quality of exposure assessment, and hence impact the results of the exposure–risk relationship.

## Introduction

Radon is present everywhere in the air in varying concentrations. Epidemiological evidence of lung cancer risk from radon is based mainly on studies of male underground miners where occupational exposures were, historically, relatively high in comparison to residential indoor exposure (NRC 1988, 1999; UNSCEAR 2009). However, over the past decades, radon levels in uranium mines have decreased significantly as the result of effective radiation protection measures in workplaces. For example, the average radon concentration in Canadian uranium mines over the past decade was 111 Bq/m^3^ (average annual exposure of 0.14 WLM, assuming *F* = 0.4 and 2000 working hours per year), comparable to the average radon concentration in Canadian homes, 77 Bq/m^3^ (Chen 2017). In addition, miners’ occupational exposure to radon is limited to their working years, whereas their exposure to environmental radon everywhere occurs throughout their lifetime. Even during their limited working years, workers spend much more time at home than in workplaces. Time-activity data showed that Canadian adults spend 16 h (67% of daily time) at home, 5.1 h (21%) at work or other facilities and 2.9 h (12%) outdoors (Matz et al. 2014; Statistics Canada 2016). Therefore, for miners with the reduced occupational radon exposures more typical of recent decades, radon exposure at home could be an important component of the cumulative radon exposure over their lifetime.

Because current miners are exposed to much lower levels of radon, cohort studies with long-term follow-up of current miners may not be feasible due to lack of statistical power at such low exposures in addition to strong confounding with tobacco smoking and, inevitably, interference from residential radon exposure. Therefore, recent studies have tried to draw relevant evidence of risk at low cumulative exposures from updated historic cohorts of uranium miners, using not the entire cohorts, but subcohorts from more recent periods characterized by significantly lower exposure rates and lower cumulative exposures resulting from protective radon mitigation measures in mines (Hunter et al. 2013; Kreuzer et al. 2018; Lane et al. 2019; Rage et al. 2015; Tomasek et al. 2008; Tomasek 2012a, b). These updated studies with improved exposure assessment generally point to higher risks for radon-induced lung cancer than previously estimated with higher cumulative exposures or higher exposure rates. Some results from the literature seem, however, to be confusing to the author, a nonexpert in epidemiology. Since radon is the largest single contributor to the natural radiation exposure to the general public and also in most workplaces including underground mines, it is of great public interest to better understand the risk of radon exposure, especially when the risk has been reassessed. Therefore, this paper is trying to share what seems controversial to the author and wondering whether lifetime exposure at home to widely varying radon concentrations can actually impact the quality of relevant exposure assessment, and hence impact the results of the exposure–risk relationship.

## Review and commentary

Epidemiological studies are conducted using models that describe lung cancer risk relative to background or reference rates. Traditionally, the comparison is made with external population background rates and, normally, the general male population is used for miner studies. Alternatively, recent cohort studies use internal comparison where the reference is not the general male population, but an internal estimate of background lung cancers at zero occupational exposure within the same cohort. As indicated in BEIR IV (NRC 1988), reliance on internal comparisons has the advantage of avoiding potential biases due to differences, other than occupational exposure of interest, between the cohort and the comparison population. It is then expected that internal comparison can remove interferences from other risk factors including residential radon exposure if, ideally, the radon distribution characteristics are the same in homes of miners at zero exposure and of miners having above-zero exposures.

Applying a stratified external approach which makes use of age-specific mortality in the general male population as the baseline, radon-associated lung cancer risk was assessed in French and Czech uranium miners at low exposure rates (generally < 7 WL) (Tomasek et al. 2008). Miners from these two cohorts are characterized by low levels of exposure (average cumulative exposure of less than 60 WLM) over a long period (mean duration of exposure is 10 years) and by good quality estimates of individual exposure (95% of annual exposures based on radon measurements). Cohort information, exposure characteristics and risk estimates are listed in Table 1.

**Table 1:** Cohort information, exposure characteristics and risk estimates (Tomasek et al. 2008)

Characteristics	Czech		French	
Follow-up period	1956–1995		1946–1994	
Cohort size	5002		5098	
Age at end of follow-up: mean (min, max)	53.7	(38, 90)	53.8	(27, 99)
Age at dead: mean (min, max)	59.5	(21, 84)	59.2	(21, 85)
Alive at end of follow-up	2977	59.5%	3792	74.4%
Lost to follow-up	137	2.7%	117	2.3%
Dead	1863	37.2%	1162	22.8%
Dead from lung cancer	449	9.0%	125	2.5%
Exposure to radon: mean (min, max)				
Age at first exposure (years)	28.6	(13, 63)	29.1	(15, 65)
Time since first exposure (years)	27.1	(4, 44)	26.7	(1, 49)
Duration of exposure (years)	9.1	(4, 37)	11.5	(1, 37)
Cumulated exposure (WLM)	57.3	(0.3, 387)	36.5	(0.1, 960)
Risk estimate (external approach)				
ERR/100WLM (overall)	3.3	95% CI 1.7–7.3	0.7	95% CI 0.2–1.5
ERR/100WLM (measured)	3.4	95% CI 1.8–7.6	2.4	95% CI 1.2–4.8
ERR/100WLM (estimated)	2.2	95% CI 0.0–5.5	0.3	95% CI − 0.2–0.8

The two cohorts are of similar size, the miners in both cohorts were subject to similar exposures and employment conditions, yet the overall risk estimate (ERR/100WLM) for Czech miners was about five times higher than that for French miners. Even though estimated risks can vary depending on the quality and size of the data used for assessment, there seems to be no reason to expect that the proportionate increase in lung cancer risk per unit radon exposure should differ so significantly by mining location.

Several factors may contribute to the large difference in estimated risks between Czech and French miners, such as systematic differences in exposure assessment and variations in smoking prevalence. A look at different radon levels in French and Czech dwellings might also help shed some light. The national average radon concentrations are 83 Bq*/*m^3^ in French homes (Billon et al. 2005) and 140 Bq/m^3^ in Czech homes (https://www.suro.cz/en/prirodnioz/rnprogram). In the Czech mining area, Bohemia, the regional average radon concentration in homes is 551 Bq/m^3^ (Tomasek 2012b), a factor of 3 higher than the national average. Compared to the general public (exposed to 140 Bq/m^3^ on average), Czech miners are, on average, exposed to 411 Bq/m^3^ more radon at home in addition to their occupational radon exposure. Because an external approach was used in the determination of exposure–risk relationship, all exposure in excess of the exposure for the reference population (140 Bq/m^3^ for the Czech public) should be considered in exposure assessment. To illustrate this point, we consider following very rough adjustment in cumulative exposure relevant to the objective of the exposure–risk study, assuming the mean age at assessment was 56 years for Czech miners. Compared to the reference population, miners were exposed to 411 Bq/m^3^ above the national average over the 56 years living in Bohemian homes, and accumulated additional exposure of 101.3 WLM (assuming 7000 h per year at home with an equilibrium factor of 0.4), about twice the occupational exposure of 57.3 WLM. In the external analysis of exposure–risk relationship, the exposure should be the total exposure for each individual miner received in mines and at home in excess of the reference population, i.e. on average a total of 158.6 WLM (57.3 WLM + 101.3 WLM). It is well known that radon concentrations in homes vary widely at any given geographical location. Even though it is not possible to retrospectively link individual occupational exposure with individual home exposure for members of these cohorts, a very rough adjustment (without considering various modifying factors included in the risk model) can reduce the estimated risk in ERR/100WLM from 3.3 to 1.2 when the total average cumulated exposure (in mine and at home in excess of the exposure to the reference population, i.e. 158.6 WLM) be used instead of only the cumulated exposure in mine (i.e. 57.3 WLM), as shown in Table 2. Accordingly, the difference of estimated risks between Czech and French miners decreases from 4.7 (3.3/0.7) to 1.7 (1.2/0.7). Most residential radon exposure–risk relationship studies focused on radon exposure at homes during the period 5–34 years prior to diagnosis of lung cancer, such as European and North American pooled studies (Darby et al. 2005; Krewski et al. 2005). If only 30-year radon exposure at home is assumed to be attributable for excess lung cancer cases, the roughly adjusted risk is 1.7 ERR/100WLM. Even though this is not an appropriate adjustment in terms of epidemiological methodology based on person-year tables and there is no consideration for any modifying effects, this example demonstrates, at least, the potential importance of residential radon exposure in exposure–risk relationship studies when residential radon levels differ significantly between miners and the reference population. The roughly adjusted risks of 1.2 or 1.7 ERR/100WLM are surprisingly closer to the excess relative risk of 1.5 per 100 WLM from a case–control study among Czech uranium miners (Tomasek 2011).

**Table 2:** Roughly adjusted risk estimate for Czech miners with consideration of total radon exposure in mines and at homes of 411 Bq/m^3^ above national average radon concentration indoors for the study using external analysis approach

Exposure to radon	Mines	Homes (411 Bq/m^3^)	Homes (411 Bq/m^3^)
Age at first exposure (years)	28.6	0	21
Time since first exposure (years)	27.1	56	56
Mean age at the assessment	55.7	56	56
Duration of exposure (years)	9.1	56	35 (exclude last 5 years)
Cumulated exposure (WLM)	57.3	101.3	54.3
Risk estimate (external approach)			
ERR/100WLM	3.3		
Rough adjustment			
Total cumulated exposure (WLM)_total_		158.6	111.6
Roughly adjusted ERR/100WLM		1.2	1.7

In the recent years almost all results from miner cohorts have been derived from internal analysis for the exposure–risk relationship where the reference is not an external general male population, but an internal population within the same cohort at zero occupational exposure, assuming all workers at zero and above-zero exposures were exposed to the similar levels of other risk factors including radon at homes. Unlike the studies using external analysis reviewed above, the newer approach of internal comparison is expected to avoid potential biases (other risk factors including radon exposure at home) due to differences, other than occupational exposure of interest, between the cohort and the comparison population. Several recent studies with internal analysis reported a significant increase in the risk when comparing the subcohort having lower cumulative exposure with the entire cohort of significantly higher cumulative exposure (Hunter et al. 2013; Lane et al. 2019; Rage et al. 2015; Tomasek 2012b). The public has been informed that newly assessed radon risk is higher than previously estimated. Two examples are shown in Table 3.

**Table 3:** French and Canadian studies for entire cohort and subcohort of lower cumulative exposures

	French	French post-55 subcohort	Beaverlodge	Beaverlodge subcohort (< 100 WLM)
Follow-up period	1946–2007	1956–2007	1950–1999	1965–1999
Cohort size	5086	3377	10 050	
Person-years at risk	179,955	110,548	150,964	134,113
Employment period	1946–1997	1955–1997	1948–1982	1965–1982
Inclusion criteria	Employed at least 1 year	Employed at least 1 year	Employed at least a day	Employed at least a day
Age at entry into study (years)	28.8 (16–68)	28.3 (17–58)	28.8	
Age at end into study (years)	64.2 (20–85)	61.1 (20–85)	Not available	
Duration of exposure (years)	13.1 (1–38)	12.9 (1–35)	1.25	
Dead from lung cancer	211	94	279	123
Cumulated exposure (WLM)	36.6 (0.01–960)	17.8 (0.01–128)	84.8	32.3
Lung cancer per 10,000 person-years per WLM	0.32	0.48	0.22	0.28
Risk estimate (internal approach)				
ERR/100WLM	0.71 (95% CI 0.31–1.30)	2.42 (95% CI 0.90–5.14)	0.96 (95% CI 0.56–1.56)	2.24 (95% CI 0.9–4.7)
Reference	Rage et al. (2015)	Rage et al. (2015)	Howe et al. (1986), Lane et al. (2010)	Lane et al. (2019)

In French mines, the assessment of exposure to radon and its short-lived decay products has changed over the years (Allodji et al. 2012). From 1946 to 1955, exposure assessment was based on retrospectively reconstructed doses. Radiation protection measures were set up in 1956, and radon exposures then individually assessed and recorded. The average cumulative exposure for monitored workers post-1955 was 17.8 WLM, significantly lower than the exposures estimated for earlier years and about half the value for the entire cohort. Lung cancer per 10,000 person-years per WLM was 0.48 for post-1955 subcohort and 0.32 for the entire French cohort. While lung cancers per 10,000 person-years per WLM for the low exposure subcohort was only about 50% higher than that for the entire cohort (i.e. 0.48 vs. 0.32), the estimated risks in ERR/100WLM differed by a factor of 3 between the entire cohort and the subcohort of lower exposures. This seems confusing to the author. Consistent with a smaller dataset, it is noticed that the 95% confidence intervals of the internal risk assessment for the post-55 subcohort were much wider than that for the entire French cohort, even though they are statistically significant.

In the Beaverlodge mine, radon and radon progeny measurements started in 1954 with fewer than 12 measurements per workplace per year, and continued with increasing frequency throughout the life of the mine (Howe et al. 1986). Personal exposures were assigned to underground miners in November 1966. Before 1966, individual annual exposures in WLM were estimated using the annual geometric mean radon progeny concentrations and time spent in the underground workplace. The Beaverlodge subcohort was constructed for the time period when routine radon monitoring was in place and only included miners with cumulative exposures lower than 100 WLM. The average cumulative exposure was 32.2 WLM, significantly lower than in earlier years and less than half the value of 84.8 WLM for the entire cohort. Similar to the French study, cumulative radon exposure was significantly associated with an increased risk of lung cancer mortality. While lung cancer per 10,000 person-years per WLM of 0.28 for the subcohort was comparable to 0.22 for the entire Beaverlodge cohort, the internally estimated risks in ERR/100WLM differed by more than a factor of 2 between entire cohort and subcohort of lower exposures. Again, this seems hard to comprehend. Noticed again that due to the smaller dataset, the 95% confidence interval of the risk for the subcohort was much wider, even though it is statistically significant.

In the literature, estimated risks for subcohorts with lower cumulative exposures are often significantly higher than the risks estimated for entire cohorts. A common explanation offered was that uranium mining started long before radiation protection measures were in place, and radon monitoring was not available in most workplaces in the early years. Exposure assessment based on very limited measurements or derived retrospectively by reconstruction and/or extrapolation was believed to be very uncertain for the early periods of mining. It is believed that radon exposure for the early periods was not only very uncertain but also overestimated and hence the risk underestimated. It is unclear why huge uncertainty can only cause an overestimation of exposure not randomly vary between under- and overestimation. Nevertheless, this overestimation may be true for many miner cohorts, such as the French and Beaverlodge studies, it cannot fully explain the differences in risk estimates made from studies on Czech miners (Table 4).

**Table 4:** Cohort information and risk estimates from Czech miner studies

Czech miners	West Bohemia cohort	West Bohemia subcohort (post 1952)	Czech subcohort (exposure rate < 7 WL)
Follow-up period	1952–1995	1956–1995	1956–1995
Size	4320	2552	5002
Person-years at risk	113,433	68,079	115,261
Dead from lung cancer	790	419	449
Cumulated exposure (WLM)	152	99	57.3
Risk estimate, ERR/100WLM	1.5 (95% CI 1.0–2.1)	2.3 (95% CI 0.9–3.8)	3.3 (95% CI 1.7–7.3)
Reference	Tomasek and Placek (1999)	Tomasek and Placek (1999)	Tomasek et al. (2008)

The main strength of the Czech studies is the low uncertainty of occupational exposure estimates resulting from extensive measurements since very beginning of the study (Tomasek et al. 2008; Tomasek 2012a). There were about 200 radon measurements per year and per shaft, in the early period (1949–1959) and more than 900 afterward. The number of measurements taken in different working places were proportional to the number of miners working at the locations. The concentrations of radon gas were converted to concentrations of radon decay products using measurements of equilibrium factors in mines when ventilation was not operated for 1 month (note: this may somewhat overestimate exposure). Each miner’s annual exposure to radon progeny was estimated, combining measurement data with registered employment details, including duration of underground work at different shafts and job category. Individual exposure data based on direct radon-progeny measurements in the ambient air were available after 1968. Therefore, the Czech cohort studies, either the entire cohort or subcohorts of low exposure rates or low cumulative exposures, all have high quality of occupational exposure assessment. Even so, the estimated risk for a cohort of lower cumulative exposure was higher than the estimated risk for a cohort of relatively higher cumulative exposure (see Table 4). Apparently, more accurate exposure measurement could not be the only or main reason for the risk increase with decreasing cumulative exposure and increasing follow-up years in the Czech studies.

## Discussion

Radon is a naturally occurring radioactive gaseous element that is ubiquitous in nature and is the largest single contributor to the natural radiation exposure to the general public as well as to most workers including miners. It is, therefore, of great public interest to better understand and confidently communicate the risk of radon exposure.

For a study on the exposure–risk relationship, the quality of exposure assessment directly impacts the quality of risk estimation. This paper explores another potential impact factor on the quality of exposure assessment, the lifelong exposure at home to widely varying radon concentrations. This factor has not yet been considered in epidemiological studies of uranium miners. As indicated in the BEIR VI report (NRC 1999), residential radon-progeny exposures of the miners were not considered in the data analysis and were implicitly assumed to be the same, on average, at all levels of occupational exposure. The BEIR VI report indicated also that any bias in the modelling due to ignoring non-mine exposures is likely to be small, because residential radon concentrations are generally much lower than mine concentrations. This may be true for the data available to the BEIR VI report at that time, but it is no longer the case for more recent miner cohorts, especially those selected sub-cohorts at low exposure levels.

In external analysis, general population is often chosen as the reference population. Miners can be exposed to higher concentrations of radon at home than the general population, because it is radon from the soil that accumulates in homes and radon concentrations in soil tend to be higher where uranium mines are located. With external approach, it seems to be necessary to consider the total radon exposure cumulated in mines and at homes in excess of the exposure of the reference population in order to more accurately assess the exposure–risk relationship. Otherwise, excluding residential exposure above the levels of the reference population could mean the exposure is underestimated and the risk relative to the reference population is then overestimated.

With internal approach, the contributions from residential radon exposure could be well accounted for if the internally estimated baseline at zero occupational exposure is representative enough. As in the case of the BEIR VI analysis (NRC 1999), residential radon exposure was assumed to be the same, on average, at all levels of occupational exposure regardless where miners lived and for how long. However, the reality could be far from ideal as assumed. It is well known that radon concentrations can vary geographically, seasonally and even daily. Many factors could affect radon and radon progeny concentrations in enclosed spaces, such as ventilation rate, moisture, solid particle concentration in the air and available surface area for radon progeny deposition. Therefore, radon can differ from one house to the other even in the same location. Radon can also vary in different units of a mine depending on the local working condition or environmental setting. All of those facts make it hard to imagine residential radon can have any correlation with radon in workplaces. For internal analysis, the evaluation of the risk of radon-induced lung cancer in a cohort of miners should ideally be determined from individual measurements of the cumulative radon exposure at home as well as in the mine, instead of simply assuming that all at home radon exposures are comparable. This is easy to say, but may not be easy to do. The main challenge is that environmental radon levels vary widely and good data for residential radon exposures (individual or local) are not always available for both miners and reference population.

In the published literature, most cohort studies for uranium miners were based on cumulative exposure acquired underground over a few years, decades prior to the exposure-risk analysis. While occupational radon exposure was limited to a few working years, environmental radon exposure occurs everywhere throughout their lifetime. Most studies confirmed the strong decreasing effect with time since exposure, such as the EPA radon risk model (EPA 2014) [a reasonable average of the estimates from the two BEIR VI preferred models for miners (NRC 1999)]:

$${e}_{a}=\beta \left({W}_{5-14}+0.78{W}_{15-24}+0.51{W}_{25+}\right){\varnothing }_{\mathrm{age}},$$

where age-specific excess relative risk, *e*_*a*_, is calculated as the weighted summation of three time-since-exposure windows, namely *W*_5–14_ the exposure incurred between 5 and 14 years before age *a*; *W*_15–24_ the exposure incurred between 15 and 24 years before age *a*; and *W*_25+_ the exposure incurred 25 years or more before age *a*. With assumed 5 years lag time, exposures incurred in more recent years contribute more to the risk. Therefore, for limited exposures accumulated underground several decades ago, the effectiveness of occupational exposure decreased with increasing length of the cohort follow-up. On the other hand, the unavoidable radon exposure at home could become more and more important with increasing duration of follow-up. During the most effective exposure window (between 5 and 30 years before the age at assessment, like assumed in most residential radon studies), cumulative exposure at home could then dominate the level of exposure, which in turn contribute to radon-induced lung cancer. Therefore, when residential radon levels differ significantly between miners and the reference population (either external or internal reference population), simply excluding information on residential radon exposure (especially the exposure occurred between 5 and 30 years before the age at assessment) could potentially impact the quality of exposure assessment, which then directly impacts the quality of the risk assessment. The lower radon levels in mines or the higher radon levels at home, and the longer time-since-exposure or the longer the follow-up years of miner cohorts, the more important it is to carefully deal with potential impact on the quality of exposure assessment resulting from widely varying radon exposure at home.

Although we can now have high-quality radon dosimetry and accurate employment records for individual miners, they may not be good enough to improve the quality of exposure assessment in the study of exposure–risk relationship. To improve the situation, we would need, ideally, high-quality radon measurement and accurate exposure record keeping for both workplaces and residential homes. Of course, this is very challenging, may not be even practical. However, it may be worth of trying to consider new means of grouping or selecting subcohorts based on miners’ residential history, at least the geographical location history if national or local radon potential map is available.

Radon is believed to be the second leading cause of lung cancer after tobacco smoking (NRC 1999; WHO 2009). In addition to tobacco smoking and radon exposure, there may well be other environmental factors also contributing significantly to the development of lung cancer. Compared to other types of cancer, lung cancer baseline risk is high and increases non-linearly with age, especially before the age of 60. The characteristics of lung cancer risks reflect the biological nature of how our bodies respond to various environmental contaminants, and also complicate the radon risk assessment of historic cohorts of uranium miners with longer follow-up years. The extended studies of uranium miners based on more lung cancer cases and longer follow-up years could allow more reliable estimate of the exposure–response relationship and various modifying effects. However, the biological effect of residential radon cannot be distinguished from the biological effect of radon in workplaces. For an exposure–risk relationship study, the excess risk is assessed based on the excess exposure of the cohort relative to the reference. Therefore, in a study of former uranium miners, excess radon-induced lung cancer cases should be related to the combined radon exposure cumulated in workplaces and at homes in excess of the radon exposure of the reference population. This is especially important when residential radon levels differ or vary significantly between miners and the reference population over the course of extended follow-up years.
